# The Evaluation of Torrefied Wood Using a Cone Calorimeter

**DOI:** 10.3390/polym13111748

**Published:** 2021-05-27

**Authors:** Peter Rantuch, Jozef Martinka, Aleš Ház

**Affiliations:** 1 Faculty of Materials Science and Technology in Trnava, Slovak University of Technology in Bratislava, 917 24 Trnava, Slovakia; jozef.martinka@stuba.sk; 2 Faculty of Chemical and Food Technology, Slovak University of Technology in Bratislava, 812 37 Bratislava, Slovakia; ales.haz@stuba.sk

**Keywords:** torrefied wood, fuel, combustion, heat release rate

## Abstract

This study focuses on the energy potential and combustion process of torrefied wood. Samples were prepared through the torrefaction of five types of wood: Ash, beech, oak, pine and spruce. These were heated for 2 h at a temperature of 300 °C under a nitrogen atmosphere. Torrefied wood was prepared from wood samples with dimensions of 100 × 100 × 20 mm^3^. These dimensions have enabled investigation of torrefied wood combustion in compact form. The effect of the external heat flux on the combustion of the samples was measured using a cone calorimeter. The observed parameters, include initiation times, heat release rate and combustion efficiency. The results show that increasing the external heat flux decreases the evenness of combustion of torrefied wood. At the same time, it increases the combustion efficiency, which reached an average value of approximately 72% at 20 kW m^−2^, 81% at 30 kW m^−2^ and 90% at 40 kW m^−2^. The calculated values of critical heat flux of the individual samples ranged from 4.67 kW m^−2^ to 15.2 kW m^−2^, the thermal response parameter ranged from 134 kW s^0.5^ m^−2^ to 297 kW s^0.5^ m^−2^ and calculated ignition temperature ranged from 277 °C to 452 °C. Obtained results are useful both for energy production field and for fire safety risk assessment of stored torrefied wood.

## 1. Introduction

The current way in which natural fossil resources are consumed to provide energy does not reflect the concept of sustainability [1]. Sustainable development is development that meets the needs of the present without compromising the ability of future generations to meet their own needs [2]. Therefore, the importance of renewable energy sources is growing. One of the possible solutions may be a more efficient use of biomass. It is a primary source of renewable carbon that can be utilised as a feedstock for biofuels or biochemical production in order to achieve energy independence [3].

In 2015, the worldwide total primary energy supply was 13,647 Mtoe, of which 13.4%, or 1823 Mtoe, came from renewable energy sources. Due to its widespread non-commercial use in developing countries, solid biofuels/charcoal remains the largest renewable energy source, representing 63.7% of the global renewable supply [4]. Torrefied wood is a fuel with the potential to partially replace coal [5].

Torrefaction is a pyrolysis process carried out at a temperature range of 200 to 300 °C under an inert atmosphere, which produces a high-quality solid biofuel that can be used for combustion and gasification [3,6,7]. It removes moisture and low weight organic volatile components and depolymerises the long polysaccharide chains, producing a hydrophobic solid product with an increased energy density (on a mass basis) and greatly increased grindability [8].

Hemicellulose, cellulose, and lignin are the basic constituents of a biomass and their thermal behaviour is highly related to the degradation of the biomass in a high-temperature environment. Biomass with torrefaction temperatures of 200 to 225 °C are described as light torrefaction; 250 °C as mild torrefaction, and 275 to 300 °C belong to severe torrefaction [6].

Based on the thermal analysis results Chen and Kuo stated that xylan is always sensitive to torrefaction in the temperature range of 200 to 300 °C. As the torrefaction temperature is no higher than 225 °C, weight loss of hemicellulose is very low. This temperature, thus, plays no part in thermal degradation of hemicellulose. Thermal degradation of cellulose is slight if the torrefaction temperature is less than or equal to 250 °C [6]. By decomposing the reactive hemicellulose fraction, a fuel with increased energy density is produced. [9] Simultaneously part of oxygen is removed from biomass [7]. During the process of wood torrefaction and with increasing temperature and time of exposure, the amount of fixed carbon, lignin and carbon in the end product also rises, with temperature as the most important factor [10]. The liquid yield is also increased [11]. According to Wannapeera and Worasuwannarak torrefaction conditions have impact on the elemental composition of torrefied wood only at a higher mass yield (>80%). Energy yield decreases with increasing degree of torrefaction [12].

The main gaseous products of the torrefied biomass combustion process are CO_2_ and H_2_O which confirms that carbon and hydrogen are significant compounds in torrefied biomass. The amount of gas decreases with increasing torrefaction temperature, probably because of gas removal during the torrefaction process [13]. Torrefied biomass has a higher pyrolysis and combustion temperature due to moisture and volatiles removal and thermal decomposition of its main components. Torrefaction also increases ash content and C/H and C/O ratio of biomass [14]. The increase in ash content of torrefied biomass is mainly due to mass loss during torrefaction reaction [15]. The lower O/C and H/C ratio is due to removal of water and carbon dioxide [16]. Moisure absorption of torrefied wood is significantly reduced due to loss of hydroxyl groups [15]. In 2019 there were produced 431 4342 t wastes from wood processing and the production of paper, cardboard, pulp, panels and furniture in the Slovak republic [17]. Although, this waste can be used for the production of other materials, such as eco-friendly, high-density fiberboards [18], 42.7% of this amount, was incinerated with energy recovery [16]. The most harvested wood in the Slovak Republic is spruce, followed by beech, fir and oak [19]. Based on data from 2019, beech (34.2%), spruce (22.1%), oak (10.5%) and pine (6.6%) have the highest proportion in forests in the Slovak Republic [20]. In relation to logging and tree species proportion, beech, oak, spruce and pine were selected as samples. Ash was chosen as a representative of less common species.

Although the torrefaction process has been used for a long time, it is still one of the important energy recovery options for biomass waste. In contrast to most previous works, torrefied wood was produced from bigger samples, not from disintegrated wood (this research represents the border between laboratory and medium-sized experiments). The burning of dust particles is significantly different from the burning of compact material. For example, in the case of dust cloud, explosive combustion can occur [21]. Our approach allowed the investigation of torrefied wood combustion in compact form, in the contrast to previously published works. The literature also lacks a description of torrefied wood in terms of its fire safety during storage. In these cases, it may be an additional fuel and may result in an increase of heat release rate during a fire. This factor subsequently affects the load-bearing capacity and integrity of the surrounding structures. Measurements using a conical calorimeter are suitable for such assessment of materials [22,23]. The aim of this article is to assess torrefied wood prepared from different woods using a cone calorimeter. The obtained results can be used in terms of energy recovery or for the needs of fire protection during storage.

## 2. Materials and Methods

**Figure 1:**
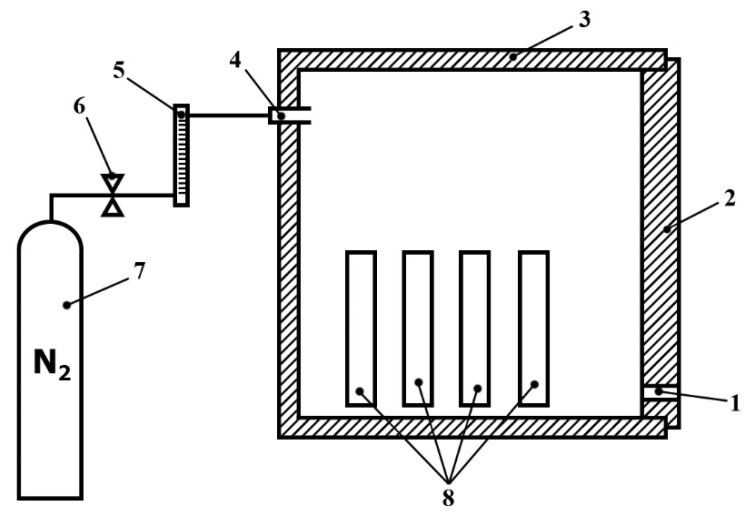
The schematic of the sample preparation device: 1—gas exhaust; 2—furnace door; 3—muffle furnace; 4—nitrogen supply; 5—volumetric flow meter; 6—reducing valve; 7—nitrogen supply tank; 8—samples.

Samples of five types of wood were selected for the preparation of torrefied wood. These included the wood of three deciduous trees: ash (*Fraxinus excelsior*), beech (*Fagus sylvatica*) and oak (*Quercus petraea*); and two coniferous trees: Spruce (*Picea abies*) and pine (*Pinus radiata*). The samples were cut tangentially into pieces with dimensions of 100 mm × 100 mm and 20 mm width. The schematic of the sample preparation device is shown in Figure 1. The torrefaction process was based on a method indicated by Liu et al. for the torrefaction of bamboo [24]. Nitrogen was used as the protective gas. It was continuously supplied to the muffle furnaceat a flow rate of 500 mL min^−1^. The samples of wood were dried at 105 °C for 24 h, and then they were inserted into the heated Nabertherm Muffle Furnace L24/11/P330 (Nabetherm GmbH, Bremen, Germany) with the temperature set at 300 °C. The residence time was 2 h. After torrefaction, the samples were placed into a desiccator, where they cooled to the ambient temperature.

The samples prepared were subsequently characterised by their proximate and ultimate analyses. Volatile matter was determined according to EN ISO 18123 [25] and ash content was measured in compliance with EN ISO 18122 [26]. Fixed carbon was calculated according to:

(1) FC=100−(VM+A)

where *FC* is fixed carbon content, *VM* is volatile matter content, and *A* is ash content.

Grounded and homogenized samples of torrefied wood were analysed (ultimate analysis) by the ELEMENTAR varioMACROcube instrument (Elementar Analysensysteme, Hanau, Alemanha). Ground and homogenization of samples were performed by Grindomix GM 200 knife mill (Retsch GmbH, Haan, Germany) at speed 10,000 min^−1^ during 10 s.

The higher heating values of the samples were measured by the IKA C4000 (IKA Analysentechnik, Heitersheim, Germany) adiabatic calorimeter. 

An important indicator of torrefaction is the energy yield, which indicates how much energy remains in the samples. Applying the relationship indicated in the work of Bach and Skrieberg, the energy yield of torrefied wood may be calculated as follows [27]:

(2) $$Y_{E}=\frac{m_{torrefied}}{m_{raw}}\times \frac{HHV_{torrefied}}{HHV_{raw}}\times 100\%$$

where $m_{torrefied}$ is the mass of torrefied wood (kg), $m_{raw}$ is the mass of raw wood (kg), $HHV_{torrefied}$ is the higher heating value of torrefied wood (MJ/kg) and $HHV_{torrefied}$ is the higher heating value of raw wood (MJ/kg).

**Figure 2:**
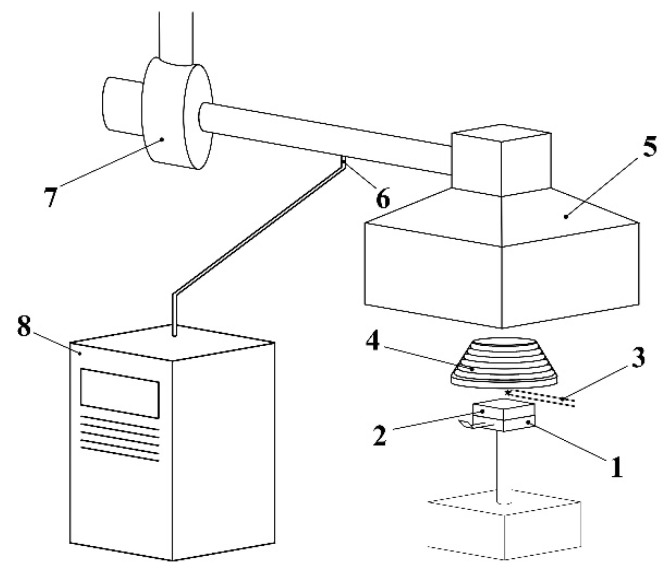
The schematic of the cone calorimeter: 1—sample holder, 2—sample, 3—initiator, 4—cone heater, 5—exhaust hood, 6—combustion gas sample extraction, 7—fan, 8—combustion gas analyser.

The measurements were carried out using a cone calorimeter (Figure 2) according to ISO 5660-1 [28]. The sample (2) was covered by aluminium foil on the surfaces that had not been exposed to the heat flux and were inserted into the holder (1). The holder was subsequently placed underneath the cone heater (4). The combustion gases were exhausted via an exhaust hood (5), with the rate of the exhaust of the thermal decomposition products regulated by adjustment of the fan (7). The extraction tube contained a circular perforated probe (6), through which the combustion gases were sampled and analysed in the CO, CO_2_ and O_2_ analysers (8).

The fan flow rate was set to 0.024 ± 0.002 m^3^ s^−1^, ambient temperature ranged from 22 °C to 27 °C and the relative humidity of air was 20–27%. The atmospheric pressure was between 100.92–101.91 kPa. Measurements were performed at heat fluxes of 20 kW m^−2^, 30 kW m^−2^ and 40 kW m^−2^. Sampling interval was set to 5 s and grinding time was 1800 s.

Fuel quality is expressed by the combustion efficiency. According to Ferek et al. the combustion efficiency of biomass can be calculated by the Equation (3) [29]:

(3) $$CE=\frac{\frac{{\left[C\right]}_{\mathrm{CO}2}}{\left({\left[C\right]}_{\mathrm{CO}2}+{\left[C\right]}_{\mathrm{CO}}\right)}-0.18}{0.82}$$

where CE is the combustion efficiency, ${\left[C\right]}_{\mathrm{CO}2}$ is the carbon emitted as CO_2_, and ${\left[C\right]}_{\mathrm{CO}}$ is the carbon emitted as CO.

The relationship characterising the time necessary for initiation can be written as [30]:

(4) $$t_{i}=\frac{\pi }{4}k\rho c{\left(\frac{T_{i}-T_{0}}{q_{e}}\right)}^{2}$$

where *t_i_* is the time to ignition, k is thermal conductivity, ρ is density, c is heat capacity, $T_{i}$ is ignition temperature, $T_{0}$ is ambient temperature, and *q_e_* is external heat flux. This equation may be adjusted as follows:

(5) $$TRP=\left(T_{i}-T_{0}\right)k\rho c$$

(6) $$\sqrt{\frac{1}{t_{i}}}=\frac{2}{\sqrt{\pi }}\frac{q_{e}}{TRP}$$

where TRP is the thermal response parameter. According to Xu et al., TRP is used as an indicator of the ignition resistance of a material [31].

Hence, the critical heat flux ($q_{cr}$) is calculated as [32]:

(7) $$\frac{q_{i}}{\;q_{cr}}=0.76$$

where $q_{i}$ is the external heat flux with an infinite time necessary for initiation.

Therefore, the formula $\sqrt{\frac{1}{t_{i}}}$ of $q_{e}$ allows the identification of the value of critical heat flux and the thermal response parameter. An advantage of the TRP calculation by this method is that no data concerning density, heat capacity and thermal conductivity at moment of ignition are needed.

Using the Stefan-Boltzman law, it is possible to conclude that:

(8) $$T_{ig}=\sqrt[{4}]{\left(\frac{\alpha q_{cr}}{\sigma }+T_{\infty }{}^{4}\right)}$$

where $T_{ig}$ is the ignition temperature at the critical heat flux and α is absorptivity, which is equal to emissivity.

Impact of wood species on the average HRR and combustion efficiency was evaluated by the Analysis of Variance (ANOVA) at a significance level *α* = 0.05. Wood species with statistically equal combustion efficiency were revealed by the Duncan’s test. The StatSoft STATISTICA 10 software was used for the ANOVA and Duncan’s test.

## 3. Results and Discussion

The mass of raw and torrefied samples are given in Table 1. The yield of torrefied wood represented 37.77–46.40%. The lowest value corresponds to ash and the highest to oak.

Proximate and ultimate analysis of individual torrefied wood samples (Table 2) indicates a high carbon content, largely in the form of fixed carbon. Volatile matter represent 36.78–44.66% and ash ranges from 0.44% to 1.11%. In terms of elemental composition the amount of hydrogen appears to be relatively low. Athough, when converted to the amount of substance, it exceeds the oxygen content. Nitrogen and sulfur were present in the samples in negligible amounts.

The high heating value was very similar in all torrefied wood samples (Table 3). Pine was slightly different from other types of wood. Energy yield ranged between approximately 54.5% and 66.5%. The ratio of O/C and H/C was 0.23–0.28 and 0.63–0.76, respectively.

**Figure 3:**
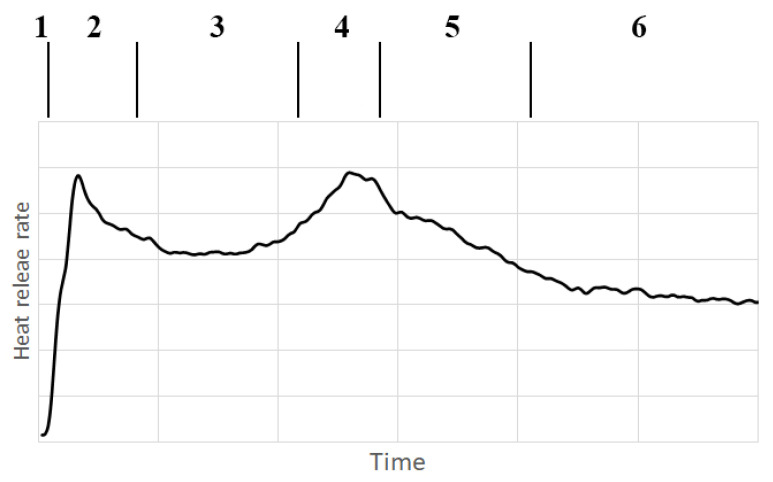
Phases of thermal degradation of torrefied wood: 1—pre-initiation phase, 2—initiation phase, 3—even combustion phase, 4—sample overheat phase, 5—low combustion phase, 6—heterogeneous combustion phase.

The cone calorimeter was used to measure the time of initiation of combustion and heat release rate (Figure 3, Table 4), as well as the overall amount of carbon oxides released, which were used to calculate the combustion efficiency for each sample (Table 5).

As to the visual comparison and rate of heat release, the process indicated in the individual charts may be divided into 6 phases.

1. The pre-initiation phase (the rate of heat release is essentially equal to zero, no visual changes in the samples can be observed),
2. The initiation phase (heat release rate rapidly increases and then falls, it is possible to see the beginning of combustion),
3. The even combustion phase (the rate of heat release is relatively constant, combustion appears even),
4. The sample overheat phase (the heat release rate increases and reaches its second peak, it is possible to observe a stronger flame),
5. The low combustion phase (the heat release rate decreases, it is possible to observe a decrease in the intensity of the flame, leading to extinction), and
6. The heterogeneous combustion phase (the speed of heat release slowly decreases; it is possible to observe blazing of the sample).

**Figure 4:**
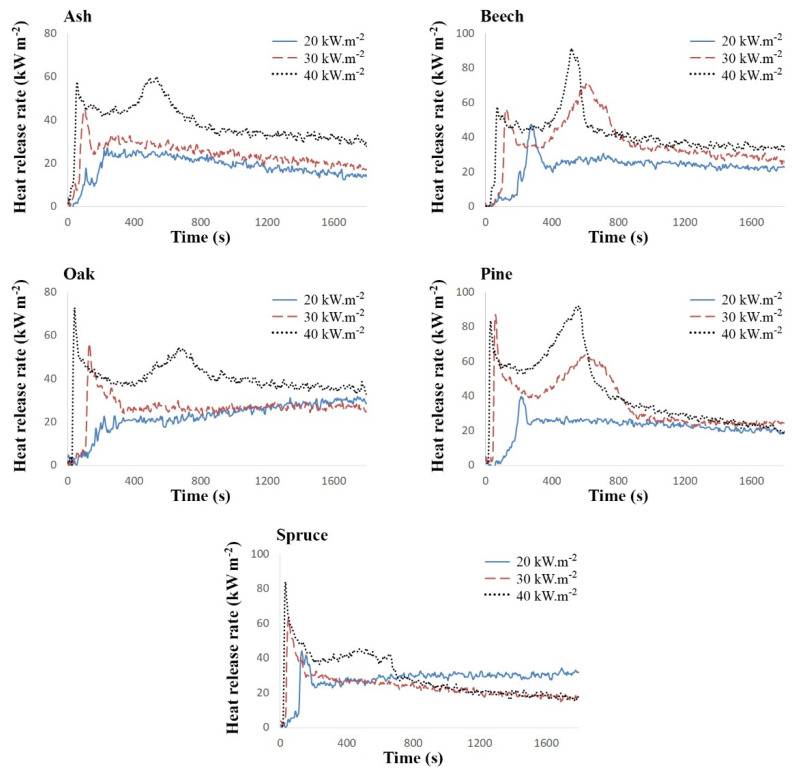
The change in heat release rate over time during the cone calorimeter measurements.

The phases are shown in Figure 3. For investigated samples of torrefied wood these phases can be recognized in Figure 4.

Heat release rate curve with two peaks is common for thermally thick materials. The first peak corresponds to the combustion of volatile combustibles before the formation of the carbonized layer [33,34] and the second is very sensitive to the thickness of the insulating substrate [35].

Impact of external heat flux and wood species on phases of thermal degradation is different. Increase of the heat flux causes greater heating of samples. This greater heating results in more clearly distinguished phases. All phases can be distinguished for all investigated samples at heat flux of 40 kW·m^−2^. On the other hand, oak, spruce and ash samples have more pronounced pre-initiation and initiation phases, while other phases are not sharply distinguished under the heat flux of 30 kW·m^−2^. Under the heat flux of 20 kW·m^−2^ only the first phase and second phase can be seen in all cases. 

**Figure 5:**
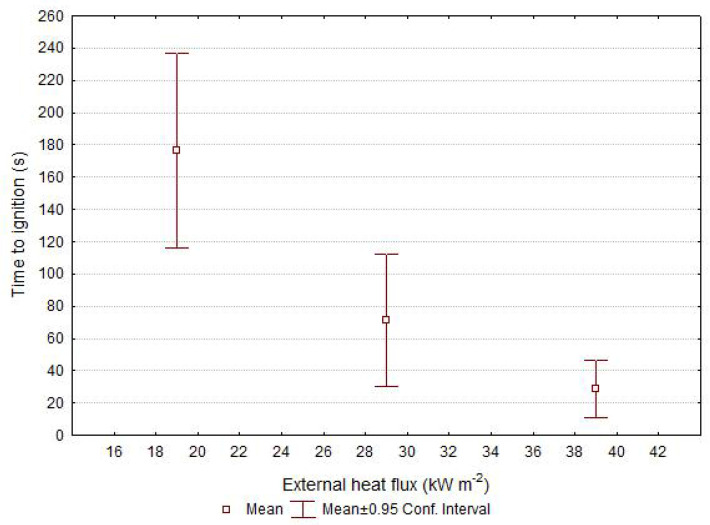
Impact of external heat flux on the time to ignition of torrefied wood.

Both the time to ignition and thus also the time duration of pre-initiation phase of torrefied wood are dependent mainly on the external heat flux (Figure 5). The heat flux radiated to the surface of the sample results in heating of the top layer of material. Heating the material to a higher temperature results in a faster release of flammable degradation products. When mixed with an oxidizing agent (mostly atmospheric oxygen), a flammable composition capable of initiation is formed. This phenomenon is well known and commonly used for ignition parameters calculation.

Time duration of initiation phase was in the range from 51 to 104 s for all samples. Impact of external heat flux on the time duration of this phase was not statistically significant.

The results clearly indicate that the higher the external heat flux, the higher the combustion rate. The heat release rate values are also higher and their peaks are shifted towards the beginning of the test.

If the external heat flux is 40 kW m^−2^, almost all the samples have two peaks. With an external heat flux of 30 kW m^−2^, the sample overheat phase is less significant, and in the case of spruce it is practically non-existent. When the samples were exposed to an external heat flux of 20 kW m^−2^, the second peak was negligible.

In general, in torrefied ash and torrefied oak, the values of released heat are almost identical to the amount of heat to which the surface of the samples is exposed. On the contrary, the highest average heat release rates were achieved by torrefied pine and torrefied beech.

ANOVA (two-way ANOVA at a significance level of *α* = 0.05) results revealing both impact of wood species and heat flux on the average heat release rate for three time intervals (300, 600 and 1200 s) are in the Table 5. The data in Table 5 proved that impact of wood species on the average heat release rate is only statistically significant for 300 and 600 s time interval. Moreover, Table 5 proved statistically significant impact of heat flux on average heat release rate for all investigated time intervals (300, 600 and 1200 s). The lowest value of the heat release rate was reached by torrefied oak. Since this type of wood has high resistance to ignition and burning even in the untreated state [36], it can be assumed that it retains similar properties compared to other woods even after the torrefaction process.

The calculated combustion efficiency values are listed in Table 6. As the external heat flux increases, so the combustion efficiency also increases, reaching, on average, less than 71% at 20 kW m^−2^, more than 81% at 30 kW m^−2^ and almost 90% at 40 kW m^−2^. The reason for the increase in combustion efficiency with increasing external heat flux is that at higher heat flux levels there is more pronounced oxidation of the solid carbonaceous layer formed on the sample during the cone calorimeter test.

ANOVA results of the impact of wood species on the combustion efficiency for investigated heat fluxes of 20, 30 and 40 kW·m^−2^ are in the Table 7. Data in the Table 7 proved that the type of wood species has statistically significant impact on the combustion efficiency.

ANOVA is able to evaluate if there are statistically significant differences between investigated samples. However, this method is not able to evaluate between which samples are significant differences. The Duncan’s test was used for this purpose. The results of the Duncan’s test are implemented to Table 6. The obtained results proved that in all investigated heat fluxes (from 20 to 40 kW·m^−2^), the difference between the pine wood and the beech wood combustion efficiency are not significant (Duncan’s test p value is higher than 0.05). At heat flux of 20 kW·m^−2^, the differences between the beech and ash wood, between spruce and ash wood and between spruce and beech wood are statistically insignificant.

By simplifying the situation and stating that the surface of torrefied wood behaves like a black body, the emissivity of torrefied wood becomes 1. The initiation temperatures calculated in this way, as well as the critical heat fluxes, the thermal response parameters and the respective determination coefficients, are indicated in Table 8.

For solution of many tasks regarding fire safety of polymers average values of ignition parameters are very important. The average values of the most important ignition parameters of torrefied wood are in the Table 9.

The yield of torrefied wood decreases with increasing temperature and time. For pine, Burgois and Guyonnet state that after 4 h at a temperature of 260 °C, it fell to 50.13%. It contained 70.71% of carbon and 24.49% of oxygen and 4.66% of hydrogen. The volatile combustible matter was 47.6% [37]. These values resemble the data that characterises the prepared torrefied wood samples. Although cited authors prepared torrefied wood at lower temperature, its influence was compensated by the longer time interval.

At 290 °C, Manouchehrinejad, van Giesen and Mani report a significantly higher volatile matter content (63.57) and a lower amount of fixed carbon (35.62) [38]. However, in the torrefaction process they used, the wood chips were exposed to an increased temperature for only 30 min. For the case of wood pellets of the torrefied wood mentioned above, the measured components are slightly closer to those of our samples.

Lee et al. also indicate that the ratio of volatile matter/fixed carbon. They report a value of 0.78 for torrefied wood pellets prepared at a temperature of 300 °C for at least 4 h, which corresponds to the values from our measurements (0.59–0.81). The carbon content (74.8%) and higher heating value (28.8 kJ g^−1^) are also similar. The hydrogen content is higher (5.1%) and the oxygen content is lower (19.2%). The energy yield is also slightly higher (69.6%) [39].

Strandberg et al. prepared torrefied wood from spruce at temperature of 310 °C during 25 min. The mass yield in the above-mentioned study (46%) was higher than mass yield from spruce prepared in this work. On the other hand energy yield published by Strandberg et al. was slightly lower (62%) than energy yield of spruce wood in this study. The elemental composition of torrified spruce wood in both studies were very similar (sample in this study contained slightly more carbon and less hydrogen and oxygen). Significant difference between torrified spruce wood was in volatile matter (51.5%) and fixed carbon (47.8%) stated in this and above-mentioned study [40]. The obtained results proved slightly higher degree of spruce wood torrefaction caused by longer duration of heat load.

Energy yield of pine wood sawdust torrefied at 300 °C for 6 min is 85.71% with higher heating value of 22.35 MJ kg^−1^ [41]. Similar to [41] the degree of torrefaction is much lower than in the case of torrefied pine at 300 ° C for 120 min due to the short exposure time of wood to high temperature.

Magdiarz, Wilk and Straka prepared (by torrefaction of fuel wood at temperature of 290 °C during 60 min) product that contains: 62.5–66.4% of carbon and 4.48–4.56% of hydrogen. Calorific value of this product was 24.4 MJ kg^−1^–26.2 MJ kg^−1^. Mass yield and energy yield were 39–43%, and 58–61%, respectively [13]. These values are almost the same as values obtained in this study. Although, the cited authors used a shorter time period in thermal loading, they prepared very similar product (the cause was the use of lower sized samples in the cited paper).

**Figure 6:**
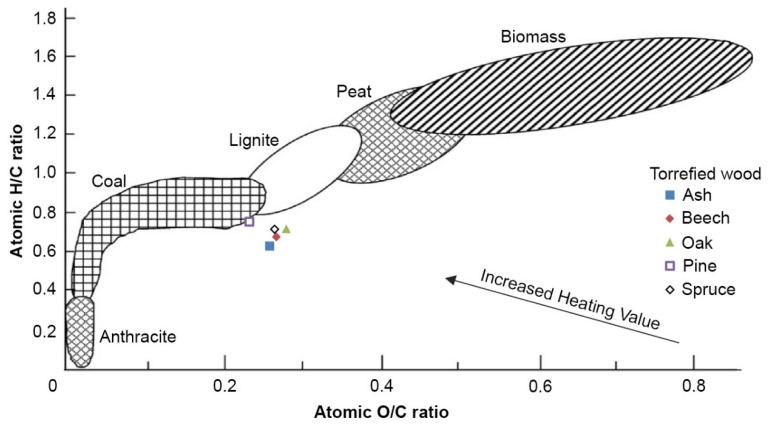
Graphic representation of the tested samples in a Van Krevelen diagram (based on Prins et al. [44]).

Solid fuels are always characterised based on their elementary H/C/O balances. A Van Krevelen diagram shows that there is a clear increase in the heating value of the different solid fuels by increasing the H/C and decreasing the O/C ratios [42]. The ranking of the results of the torrified wood samples compared to other fuels is shown in Figure 6. Torrefied pine clearly has similar features to coal. Similarly, Elaieb et al. described the charcoal produced by carbonization at a temperature of 550 °C over the course of 6 h [43].

As to safe storage, it is necessary to evaluate the ability of the individual materials to contribute to the ignition and spread of fire. It is important to know their reactions to sources of radiant heat, which include both hot surfaces (e.g., heaters) and flame re-radiation. The cone calorimeter measurements were used for this purpose. As mentioned above, there were two peaks in the measurement of the heat release rate. It is well-known that the first peak is linked to the combustion ignition. The second one was recorded at the end of the measurements. This process is also typical of untreated wood. When a sample of finite thickness is burned in a heat release calorimeter, the HRR increases toward the end of the test as a result of the near adiabatic conditions on the unexposed side [45]. The effective heat of pyrolysis is low when the thermal wave reaches the rear insulating surface and the original material is already preheated to the pyrolysis temperature [33].

Several authors have observed the effect of thermal treatment of the wood on the rate of heat release during combustion. Luptakova et al. states that heat treatment of wood at temperatures of 200–260 °C resulted in a lower mass loss a lower average relative burning rate, but it did not influence ignition time, the flame-out time, and maximum burning rate [46]. Based on measurements taken at external heat fluxes of 15–40 kW m^−2^ Martinka et al. state that the heat treatment of spruce causes a significant decrease in the maximum heat release rate [47]. Xing and Li. reached similar conclusions [48]. Lahtela and Kärki impregnated thermally treated wood with melamine and found that the heat treatment reduced the HRR values, but melamine impregnation before heat treatment was able to raise it to a higher value [49]. The aforementioned values of the peak heat release rates are significantly higher than for torrefied wood, which may be ascribed to the significantly lower temperatures used for the thermal treatment (180–220 °C) as opposed those used in torrefaction.

Elaieb et al. used a cone calorimeter to directly test the carbonized wood. However, they employed an oil burner as an initiator and observed the ignition time, combustion duration; combustion states and smoke [43]. For these reasons, it is impossible to compare the sets of results.

The critical heat fluxes calculated based on the initiation times, the thermal response parameters and initiation temperatures of torrefied wood resemble those stated by other authors for different types of wood (Table 10). Hence, the samples may be classified into two groups based on the calculated values of critical heat flux: Torrefied ash, torrefied beech and torrefied spruce reach values of less than 10 kW m^−2^, and torrefied oak and torrefied pine over 10 kW m^−2^. Nonetheless, for torrefied oak, the correlation coefficient of the corresponding equation is significantly lower, which is why the values of critical heat flux, heat response parameter and initiation temperature are only indicative.

## 4. Conclusions

Based on the measurements conducted on samples of torrefied wood from five different types of wood, it was discovered that the placement of such fuel in the van Krevelen chart is close to coal and lignite. Treatment at 300 °C for 2 h under nitrogen also appears to be sufficient for samples with dimensions of 100 mm × 100 mm × 20 mm with an energy yield from 49.45 to 61.09%. The samples were measured on cone calorimeter in a compact form. Therefore, the obtained results are suitable for use especially in places where torrefied wood does not occur in the crushed state.

The heat release rate increases with increasing external heat flux, although it also increases unsteadiness of combustion. Two clear peaks occur in the heat release rate at an external heat flux of 40 kW m^−2^, but these are significantly lower than those from the thermally untreated biomass. The combustion of the torrefied wood while making measurements using a cone calorimeter can be divided into 6 phases: Pre-initiation phase, the initiation phase, the even combustion phase, the sample overheat phase, the low combustion phase and the heterogeneous combustion phase.

The combustion efficiency identified based on the amount of CO and CO_2_ in the combustion gases increases as the external heat flux increases. On average, it reaches almost 71% at a heat flux of 20 kW m^−2^, more than 81% at 30 kW m^−2^ and almost 91% at 40 kW m^−2^.

Torrefied wood increases the fire load of fire compartments during storage (in comparison with unmodified wood). The obtained results are key for designing the fire safety of buildings where this material is stored.

## Figures and Tables

**Table 1:** The mass of samples before and after the torrefaction.

Torrefied Wood	Weight of Raw Samples (g)	Weight of Torrefied Samples (g)	Mass Yield of Torrefied Wood (%)
Ash	127.95 (±2.01)	48.31 (±2.39)	37.77 (±2.13)
Beech	131.11 (±4.28)	50.52 (±3.24)	38.51 (±1.65)
Oak	127.06 (±3.12)	58.91 (±3.30)	46.40 (±3.05)
Pine	88.28 (±3.04)	38.98 (±1.44)	44.20 (±2.30)
Spruce	81.05 (±1.88)	35.91 (±1.33)	44.30 (±1.23)

Numbers in parentheses represent standard deviation.

**Table 2:** Proximate and ultimate analysis of torrefied wood samples.

Torrefied Wood	Proximate Analysis (%)			Ultimate Analysis (%)				
	Volatile Matter	Fixed Carbon	Ash	C	H	O ^a^	N	S
Ash	36.78 (±2.68)	62.11 (±2.54)	1.11 (±0.31)	71.27 (±0.62)	3.79 (±0.07)	23.52 (±0.22)	0.22 (±0.04)	0.08 (±0.03)
Beech	41.88 (±0.34)	57.16 (±0.18)	0.96 (±0.24)	70.55 (±0.57)	4.03 (±0.07)	24.23 (±0.27)	0.23 (±0.07)	0.00 (±0.00)
Oak	42.16 (±0.35)	57.41 (±0.53)	0.44 (±0.20)	69.57 (±0.56)	4.21 (±0.09)	25.61 (±0.28)	0.16 (±0.06)	0.01 (±0.01)
Pine	44.60 (±1.67)	54.70 (±1.69)	0.70 (±0.07)	72.57 (±0.72)	4.61 (±0.08)	21.91 (±0.30)	0.19 (±0.03)	0.02 (±0.01)
Spruce	42.53 (±2.47)	56.80 (±2.26)	0.67 (±0.22)	70.59 (±0.63)	4.27 (±0.06)	24.34 (±0.25)	0.13 (±0.03)	0.00 (±0.00)

Numbers in parentheses represent standard deviation; ^a^ By calculation.

**Table 3:** The energy characteristics of samples of torrefied wood.

Torrefied Wood	HHV (kJ g^−1^)	Energy Yield (%)	Atomic O/C Ratio (-)	Atomic H/C Ratio (-)
Ash	28.79 (±0.3)	54.57 (±2.90)	0.26	0.63
Beech	28.25 (±0.7)	55.02 (±1.99)	0.27	0.68
Oak	28.18 (±0.1)	66.58 (±4.45)	0.28	0.72
Pine	30.26 (±1.0)	63.83 (±1.09)	0.23	0.76
Spruce	28.64 (±0.7)	63.22 (±1.94)	0.27	0.72

Numbers in parentheses represent standard deviation.

**Table 4:** Cone calorimeter results.

Torrefied Wood	External Heat Flux	pHRR [kW m^−2^]		Time to pHRR [s]		Time to Ignition [s]	Average HRR [kW m^−2^]		
		First	Second	First	Second		300 s	600 s	1200 s
Ash	20	27.08	-	240	-	180	23.59	23.62	21.57
	30	44.72	33.44	105	260	69	30.74	29.71	27.05
	40	57.68	60.00	55	535	35	44.92	48.37	42.43
Beech	20	47.52	30.83	275	710	246	28.67	28.18	26.53
	30	56.49	71.09	125	615	102	38.43	48.17	42.33
	40	57.79	91.54	70	515	51	46.56	54.31	46.76
Oak	20	22.82	31.74	285	1745	165	19.23	20.03	22.61
	30	56.22	29.86	130	655	107	32.79	29.72	28.08
	40	72.78	54.34	40	670	24	43.68	42.92	42.90
Pine	20	39.75	28.19	215	490	181	27.26	26.56	25.01
	30	86.86	64.12	60	605	45	47.24	50.37	42.60
	40	83.620	91.99	30	560	17	58.34	67.01	51.61
Spruce	20	44.16	34.38	130	1695	110	28.05	27.59	28.75
	30	63.43	-	50	-	33	35.07	30.76	26.73
	40	83.99	49.08	30	125	17	46.79	44.25	35.12

**Table 5:** Results of two-way ANOVA examining both the impact of wood species and heat flux on the average HRR during investigated time intervals (300, 600 and 1200 s) at a significance level *α* = 0.05.

ANOVA Coefficients	Average HRR in Time Interval [s]		
	300	600	1200
*p* (wood species)	0.0108	0.0218	0.1009
*F* (wood species)	6.8152	5.3207	2.7934
*F*_crit_ (wood species)	3.8379	3.8379	3.8379
*p* (heat flux)	1.38 × 10^−5^	0.0002	0.0009
*F* (heat flux)	61.62	28.65	18.65
*F*_crit_ (heat flux)	4.4589	4.4589	4.4589

**Table 6:** Combustion efficiencies of torrefied wood obtained using different heating fluxes.

Torrefied Wood	Combustion Efficiency [%]		
	q_e_ = 20 kW m^−2^	q_e_ = 30 kW m^−2^	q_e_ = 40 kW m^−2^
Ash	75.1 (6.8) ^bc^	80.7 (9.5)	89.4 (5.4)
Beech	74.7 (7.2) ^abd^	87.9 (5.8) ^a^	92.1 (4.5) ^a^
Oak	61.8 (10.2)	73.1 (9.3)	88.5 (5.5)
Pine	72.3 (6.9) ^a^	87.6 (6.1) ^a^	92.2 (4.2) ^a^
Spruce	74.4 (7.0) ^cd^	77.8 (4.8)	85.7 (7.4)

Numbers in parentheses represent standard deviation, ^a^ wood species with statistically equal combustion efficiencies at all investigated heat fluxes, ^b,c,d^ wood species with statistically equal combustion efficiency at heat flux of 20 kW·m^−2^.

**Table 7:** Results of ANOVA examining the impact of wood species on the combustion efficiency for heat fluxes of 20, 30 and 40 kW·m^−2^ (at significance level *α* = 0.05).

ANOVA Coefficients	Heat flux [kW·m^−2^]		
	20	30	40
*p*	3.9 × 10^−137^	2.7 × 10^−183^	1 × 10^−66^
*F*	192.03	272.77	85.75
*F*_crit_	2.38	2.38	2.38

**Table 8:** Critical heat flux and thermal response parameter of torrefied wood.

Torrefied Wood	Critical Heat Flux [kW m^−2^]	Thermal Response Parameter [kW s^0.5^ m^−2^]	R^2^ [-]	Ignition Temperature [°C]
Ash	5.7	240	0.9997	303
Beech	4.67	297	0.9981	277
Oak	13.2	179	0.8590	428
Pine	15.2	134	0.9959	452
Spruce	8.9	152	0.9984	365

**Table 9:** Average ignition parameters of torrefied wood.

Ignition Parameter	Value ± Standard Deviation
Critical heat flux [kW·m^−2^]	9.5 ± 4.6
Thermal response parameter [kW·s^0.5^·m^−2^]	200.4 ± 67.3
Ignition temperature [°C]	365 ± 76

**Table 10:** Critical heat fluxes and thermal response parameters of selected types of wood.

Wood	Critical Heat Flux [kW m^−2^]	Thermal Response Parameter [kW s^0.5^ m^−2^]	Ignition Temperature [°C]	Source
Douglas fir	18	182	478	[31]
Scots pine	19	164	488	[31]
Southern pine	19	201	488	[31]
Shorea	16	152	456	[31]
Merbau	40	275	643	[31]
Redwood	15.5	-	375	[32]
Red oak	108	-	304	[32]
Douglas fir	16.0	-	384	[32]
Maple	13.9	-	354	[32]
Nordic spruce	19.0	291	488	[33]
Fir	11.6–12.0	128–144	372.7	[50]
Radiata pine	13.2	-	-	[51]
Pacific maple	10.3	-	-	[51]
Sugar pine	14.0	-	-	[51]
Bamboo	6.0–8.0	235–376	297–340	[52]
Spruce	10.1	-	-	[53]
Softwood	10.0	-	-	[54]
Leadwood	15.0	376.2	-	[55]
Mopani	14.4	161.2	-	[55]
Tamboti	5.9	352.7	-	[55]
Stinkwood	9.2	173.6	-	[55]
Real Yellowwood	1.3	232.2	-	[55]
